# Poliovirus serological assay after the cVDPV1 outbreak in Papua New Guinea: a cross-sectional study from 2020 to 2021

**DOI:** 10.1016/j.lanwpc.2023.100986

**Published:** 2023-12-20

**Authors:** William Pomat, Rocio Lopez Cavestany, Visalakshi Jeyaseelan, Rebecca Ford, Janet Gare, Tigran Avagyan, Varja Grabovac, Deborah Bettels, Dessie Mekonnen, Kathryn Ann Vetter Jones, Bernardo Alfredo Mainou, Ondrej Mach

**Affiliations:** a Papua New Guinea Institute of Medical Research, Goroka, Eastern Highlands Province, Papua New Guinea; b Polio Eradication Department, World Health Organization Headquarters, Geneva, Switzerland; c World Health Organization, Western Pacific Regional Office, Manila, Philippines; d World Health Organization, Papua New Guinea Country Office, Port Moresby, National Capital District, Papua New Guinea; e US Centers for Disease Control and Prevention, Atlanta, Georgia, USA

**Keywords:** Polio, Vaccine-derived poliovirus, Bivalent oral poliovirus vaccine, Seroprevalence, Outbreak response, Papua New Guinea

## Abstract

**Background:**

In June 2018, a type 1 circulating vaccine-derived poliovirus (cVDPV1) outbreak was declared in Papua New Guinea (PNG), resulting in a total of 26 paralytic confirmed cases. Eight vaccination campaign rounds with bivalent oral poliovirus vaccine (bOPV) were carried out in response. Prevalence of neutralizing polio antibodies in children was assessed two years after the outbreak response was completed.

**Methods:**

We conducted a cross-sectional serological survey among children aged 6 months–10 years selected from six provinces in PNG to evaluate seroprevalence of neutralizing polio antibodies to the three poliovirus serotypes and analyse sociodemographic risk factors.

**Findings:**

We included 984 of 1006 enrolled children in the final analysis. The seroprevalence of neutralizing polio antibodies for serotype 1, 2 and 3 was 98.3% (95% CI: 97.4–98.9), 63.1% (95% CI: 60.1–66.1) and 95.0% (95% CI: 93.6–96.3), respectively. Children <1 year had significantly lower type 1 seroprevalence compared to older children (p < 0.001); there were no significant differences in seroprevalence among provinces.

**Interpretation:**

PNG successfully interrupted transmission of cVDPV1 with several high coverage bOPV campaigns and seroprevalence remained high after two years. The emergence of cVDPV strains underscores the importance of maintaining high levels of routine immunization coverage and effective surveillance systems for early detection.

**Funding:**

10.13039/100004423World Health Organization through a Rotary International IPPC grant.

**Research in context**

**Evidence before this study**

Outbreaks of circulating vaccine-derived polioviruses (cVDPV) may, in rare circumstances, emerge in countries with poor routine immunization coverage. Papua New Guinea (PNG) experienced a cVDPV type 1 outbreak in 2018 that resulted in 26 paralytic confirmed cases.

**Added value of this study**

We measured seroprevalence of poliovirus neutralizing antibodies in PNG two years after the outbreak and after several vaccination campaigns with bivalent oral poliovirus vaccine (bOPV) aimed at stopping poliovirus transmission. We showed high antibody seroprevalence in those children who were reached by the campaigns.

**Implications of all the available evidence**

The results of this study provide clear cut evidence that vaccination campaigns are an essential tool to rapidly increase population immunity and interrupt poliovirus transmission.

## Introduction

The Global Polio Eradication Initiative (GPEI) was established in 1988 with the goal to eradicate all types of wild poliovirus (WPV) through strengthening of routine immunization (RI), implementing supplemental vaccination activities, and rapid detection and response to combat poliovirus transmission.^1^^,^^2^ Due to its low cost, ease of administration, and immunogenic properties to induce both humoral and mucosal immune responses, oral poliovirus vaccine (OPV) has been the predominant vaccine used to eradicate poliovirus. However, in rare instances, the attenuated virus contained within OPV can genetically revert and reacquire neurovirulence after prolonged transmission and replication in under immunized populations. In this instance, vaccine-derived polioviruses can cause outbreaks through person-to-person transmission, predominantly in areas with low routine vaccination coverage. These viruses are referred to as circulating vaccine-derived polioviruses (cVDPV).^3^

In 2022, there were 30 paralytic cases caused by WPV1 globally and 880 paralytic cases caused by cVDPV (190 by type 1, 689 by type 2, and 1 by type 3).^4^^,^^5^ WPV1 transmission occurred in the two remaining endemic countries, Afghanistan and Pakistan, as well as in Mozambique due to an importation from Pakistan. The two other WPV serotypes, types 2 and 3, were certified as eradicated in 2015 and 2019, respectively. Nearly 80% of cVDPV cases were type 2, primarily concentrated in DR Congo, Yemen, and West Africa. Most cVDPV1 cases were also in DR Congo, with incidences in south-eastern Africa.

The last poliomyelitis case in Papua New Guinea (PNG) due to WPV occurred in 1996. PNG, together with the rest of the Western Pacific Region, was certified polio-free in 2000 by the World Health Organisation.^6^ Since then, acute flaccid paralysis (AFP) surveillance in PNG has significantly declined. In 2017, the annual national non-polio AFP rate was 0.8 per 100,000 children <15 years—well under the required rate of ≥2 for sufficient sensitivity.^7^ The RI schedule in PNG includes three doses of bivalent OPV (bOPV) and two doses of IPV. The first dose of IPV was added to RI schedules in 2016 and the second July 2021, which is outside the timeframe of this study. In 2018, best estimate for RI coverage of the 3rd OPV dose was 42% and 35% for first dose of IPV—one of the lowest in the world.^8^ PNG faces obstacles in delivering immunization programs due to its scattered rural populations, challenging geography (i.e., pronounced mountain ranges, archipelagos), and limited resources for transport and healthcare.^9^ Moreover, previous research has shown that children residing in hard-to-reach areas experience even greater difficulties in receiving timely and comprehensive immunization coverage.^10^ These challenges stem from transportation barriers, insufficient awareness about vaccines, and distrust in national healthcare services. All of these factors, notably a low RI coverage, predisposed PNG for a cVDPV outbreak.

**Fig. 1:**
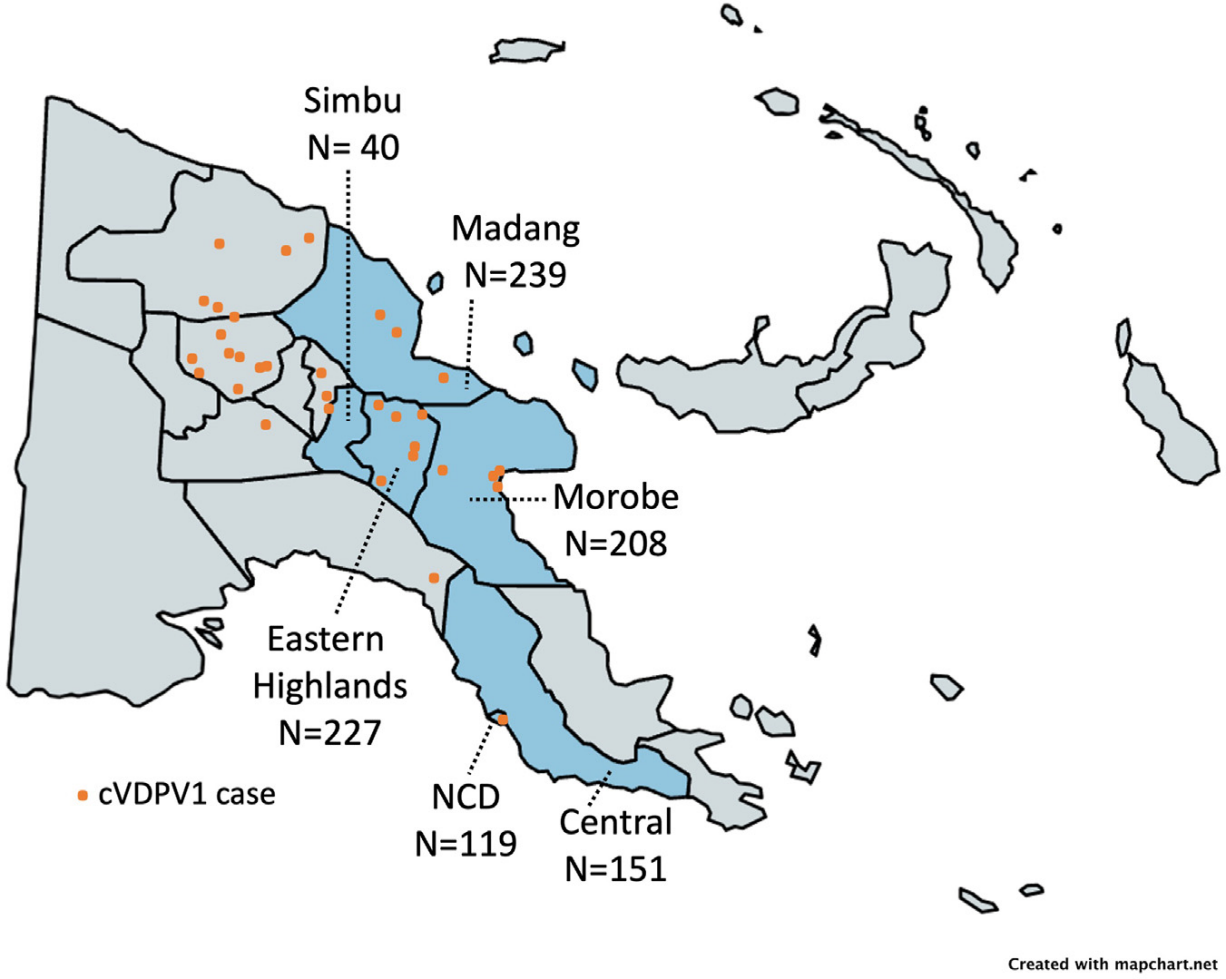
**Map of Papua New Guinea showing the provinces with cVDPV1 cases resulting from the 2018 outbreak.** The cVDPV1 reports include cases of acute flaccid paralysis and contact cases. The shaded provinces were included in this seroprevalence survey and include Simbu, Madang, Morobe, Eastern Highlands, National Capital District (NCD), and Central. The sample size enrolled from each province is indicated. cVDPV1, type 1 circulating vaccine-derived poliovirus.

In June 2018, a cVDPV1 outbreak was confirmed in PNG first detected in a 6-year-old boy from Lae, Morobe Province with acute flaccid paralysis (AFP).^11^^,^^12^ A total of 26 confirmed cases of cVDPV1 resulted from this outbreak (Fig. 1). The age group most affected were children <5 years (76% of cases), with more than half having received no OPV doses. The cases were located in 9 out of 22 provinces in PNG, primarily in areas with large transient populations, including those near mines or plantations (i.e., Eastern Highlands, Enga, East Sepik).^7^ The outbreak has largely been considered as a reflection of the poor level of routine vaccination coverage within the country, particularly in the Highlands regions.

As part of the outbreak response, polio vaccination campaigns were conducted to vaccinate children under 15 years and help to protect them against polio. Eight rounds with bOPV were conducted at national or subnational level. The administrative coverage of the rounds ranged between 70 and 100%. Active AFP case-finding at health facilities was intensified; the non-polio AFP rate increased to 7.0 in 2018.^7^ Environmental sampling was established at three sites in Port Moresby and two in Lae, effectively detecting seven cVDPV1 positive samples in 2018.

Here, we conducted a cross-sectional serological survey among children aged 6 months–10 years randomly selected from six provinces in PNG with the objective to evaluate prevalence of poliovirus-neutralizing antibodies against all three poliovirus serotypes. This survey was conducted after the outbreak response was completed.

## Methods

This was a population-based, cross-sectional serological survey conducted from April 2020 to July 2021. The study was conducted in six provinces that had been considered as high-risk for polio because they reported cVDPV1 cases, or their surveillance was considered to be sub-optimal (Fig. 1). The provinces include Madang, Morobe, Eastern Highlands, Simbu, National Capital District and Central Province. Within each province, districts were selected based on logistics and convenience. In each district, children were approached in house-to-house manner with assistance of a community elder. Households were randomly selected in consultation with the community elder. The in-country team consisted of IMR staff.

Healthy children aged 6 months to 10 years were included in the study. Exclusion criteria were existing contraindication for venepuncture and sickness requiring hospitalization. Age-groups for analysis were defined at 6 months–<1 year, 1–<5 years, and 5–10 years. The age cut-offs at 1 and 5 years are due to biological and epidemiological reasons. Within this specific study set due to the timing of sampling, children <1 year were born after the SIAs, and children >5 would have received tOPV in RI before the switch. Moreover, maternal antibodies would have completely waned after 1 year of age, and SIAs are administered in children <5 years.

To be able to compare amongst the three age groups and six provinces, 54 children were needed from each age group to provide for ±10% difference at alpha = 0.05 and beta = 0.8. To account for a possible sample loss of 10%, the number was elevated to 60 children in each age group from every province, yielding a total target sample size of 1080 children.

After informed consent was received from the child’s parent or guardian, sociodemographic characteristics were recorded (including vaccination history), and one venous blood sample (∼2 mL) was drawn and shipped to CDC Atlanta. Sera were analysed for all three serotypes of polio antibodies using a standard micro-neutralization assay.^13^ Seropositivity was defined as presence of neutralizing antibody titre in dilution ≥1:8; herein, antibody titres are presented in log_2_ scale. The maximum dilution tested was 1:1024 (and the highest detectable titre reported was 1:≥1448); the minimum (nondetectable) titre reported was <1:8. The primary outcome was seroprevalence of antibodies to poliovirus type 1, however we also report seroprevalence of serotypes 2 and 3. Significance was reported at p < 0.05.

Data were analysed using SAS (Version 9.1.3) and R (Version 4.2.2). Descriptive statistics were performed for sociodemographic variables. Seroprevalence is presented as a percent average with 95% confidence intervals. Antibody titres are presented with median and percentile 95% Bootstrap confidence intervals. The distribution of titres was compared using the log-rank test. Sociodemographic characteristics were associated with seroprevalence using Fisher’s exact test; logistic regression analysis (reporting odd’s ratio [OR] and 95% confidence interval [95% CI]) was also applied to associate vaccination history characteristics with type 1 and 3 seroprevalence. A sensitivity analysis was done to associate the vaccine history variables adjusted for confounders (Suppl 1).

The study protocol was reviewed and approved by the WHO Ethical Review Committee, PNGIMR IRB, and PNG MRAC. Scientific review was also conducted and approved by the Polio Research Committee at WHO.

### Role of the funding source

This study was funded by WHO through a Rotary International grant. WHO provided technical assistance to PNG IMR for study design, data analysis, interpretation, and writing of the report.

## Results

### Sociodemographic characteristics

A total of 1006 children were enrolled and had blood samples collected. Of those, 984 (97.8%) were eligible for final analysis; during data cleaning, the excluded children were either outside of the allowable age range (n = 22) or had missing serology data (n = 1). The children in our cohort were born between January 2010 and December 2020.

The demographic characteristics, vaccination history, water supply, sanitation and hygiene are presented in Table 1. The median age was 51 months, with most children in the 5–10-year category.

**Table 1:** Distribution of socio-demographic characteristics.

	n	%
**Total**	984	100
**Age categories**^a^		
6 months–<1 year	53	5.4
1 year–<5 years	489	49.7
5 years–10 years	442	44.9
**Age** (Median [IQR])	51	29.8–84
**Sex**		
Female	488	49.6
**Province**^b^		
Central	151	15.3
Eastern Highlands	227	23.1
Madang	239	24.3
Morobe	208	21.1
National Capital District	119	12.1
Simbu	40	4.1
**Source of drinking water**		
Protected water	140	14.3
Pipe water	285	29.0
Surface water	359	36.6
Unprotected water	198	20.2
**Type of toilet**		
Outside (Sea, Bush, pit)	968	98.5
Inside (Flush/Bucket)	15	1.5
**Handwash facility**		
Yes	888	90.4
**Breastfed**		
Yes	145	14.7
Unknown	3	0.3
No	141	14.3
**Polio supplementary vaccine (OPV)**		
Yes	913	93.1
**OPV supplementary vaccine doses received**		
1	64	7.0
2	151	16.6
≥3	696	70.7
**Vaccination records**		
Child Health Book available	683	69
**OPV routine immunization doses received**		
Full schedule (3 doses)	667	67.8
0 doses	77	7.8

a Born between January 2010 and December 2020.

b Sample dates: Central May–June 2021, Eastern Highlands April–June 2020, Madang July 2020, Morobe October 2020, NCD May–June 2021, Simbu July 2021.

In our sample, 71% of the children reported receiving 3 or more doses of OPV from SIAs and 68% reported receiving the full 3-dose schedule from RI. Vaccination records are stored in the child health book, which was available in 69% of children albeit inconsistently filled.

Most of the participants practice outdoor defecation (i.e., bush, sea, pit) and have surface water (i.e., river) as their drinking water source (98.5% and 36.6%, respectively).

### Serological analysis

**Fig. 2:**
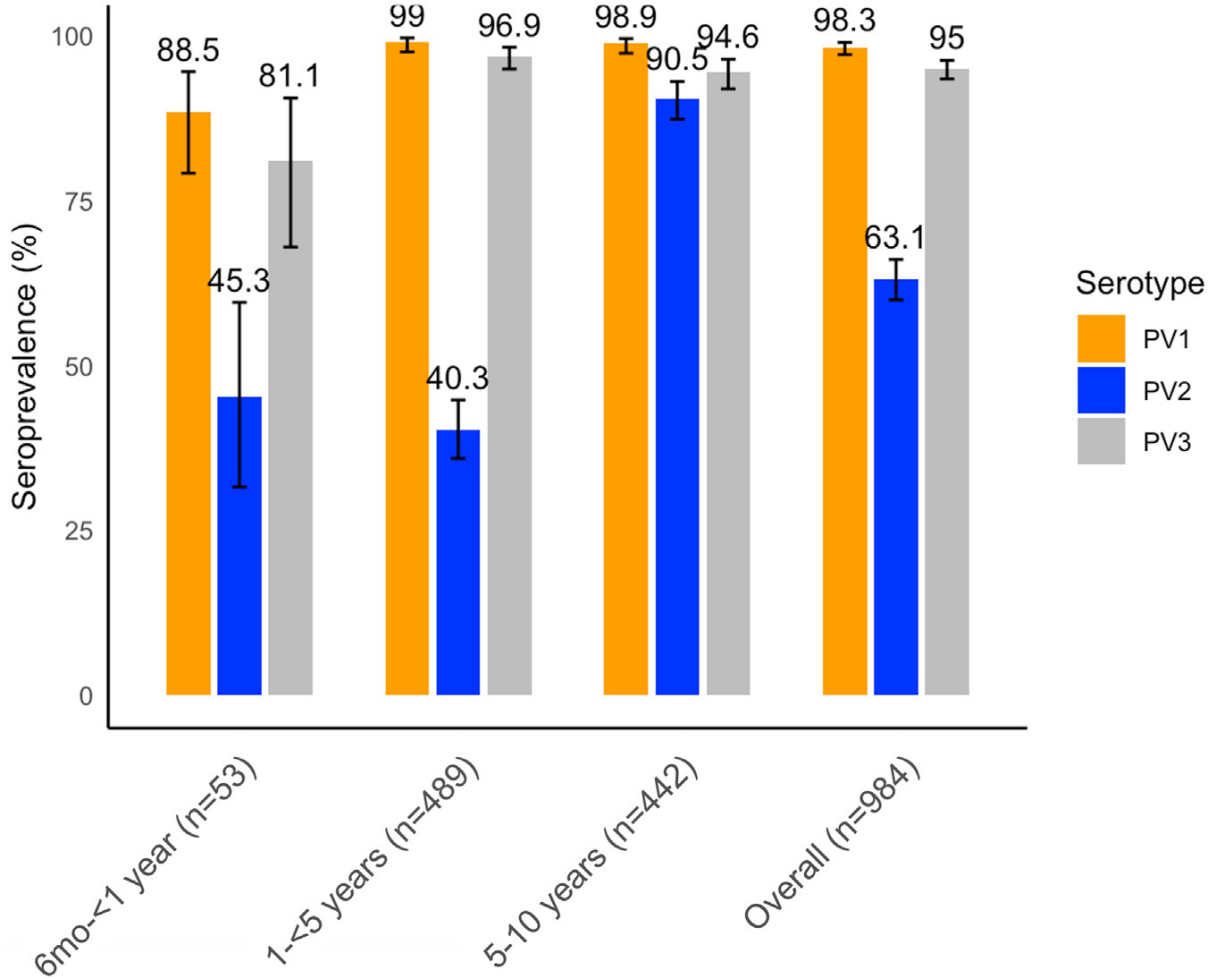
**Seroprevalences with 95% CIs by age categories.** Children born between January 2010 and December 2020. PV, poliovirus.

**Fig. 3:**
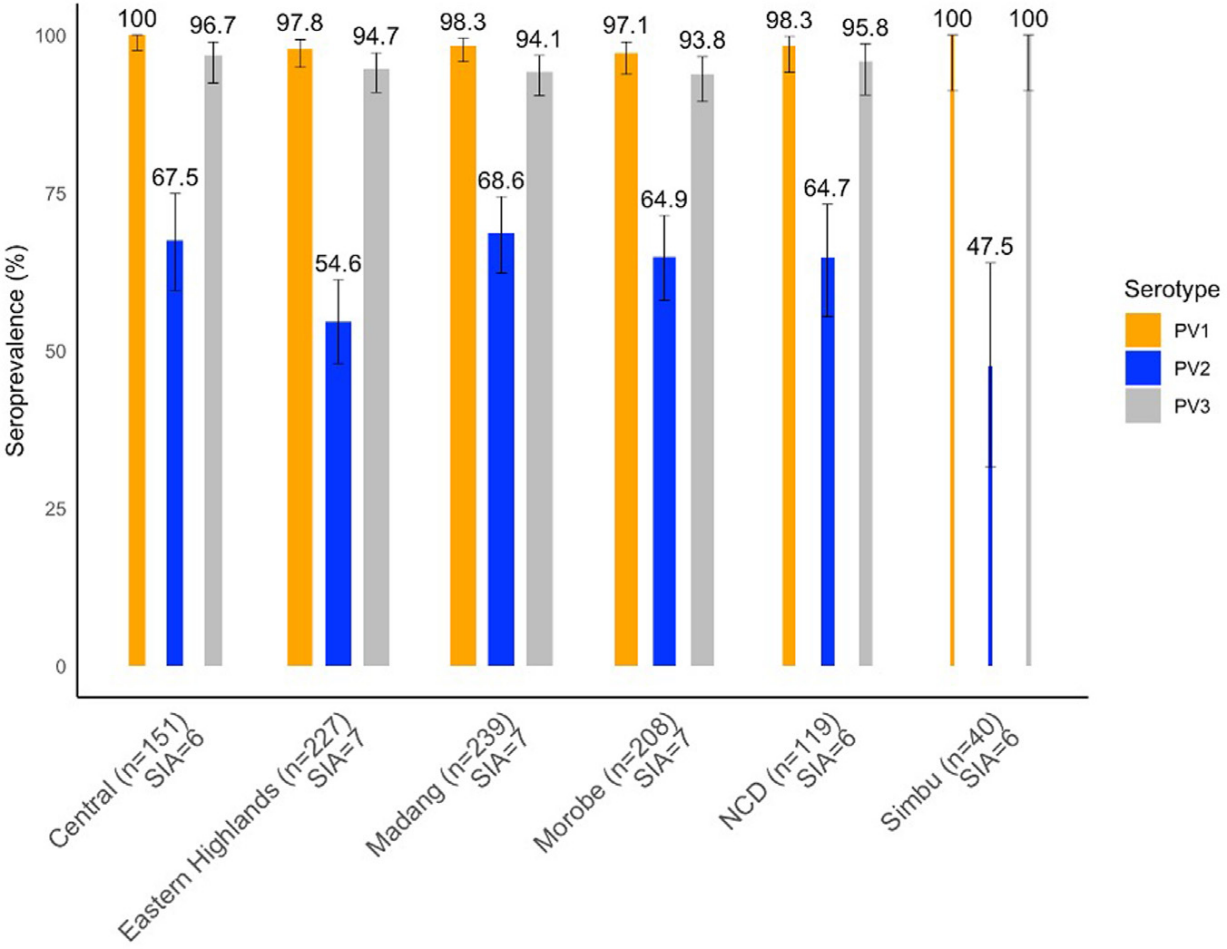
**Seroprevalences with 95% CIs by province.** Children were born between January 2010 and December 2020. The bar width is proportional to the sample size from each province. The number of bOPV SIAs conducted in each province is indicated. bOPV, bivalent oral poliovirus vaccine; PV, poliovirus; SIA, supplementary immunization activity.

The seroprevalence of neutralizing polio antibodies for serotype 1, 2 and 3 was 98.3% (95% CI: 97.4–98.9), 63.1% (95% CI: 60.1–66.1) and 95.0% (95% CI: 93.6–96.3), respectively (Fig. 2). Type 2 seroprevalence is significantly lower (p < 0.01 each, respectively). More than 90.0% of children, above the age of 1 year, were seropositive for types 1 and 3. Type 2 seroprevalence was significantly higher (90.5%, 95% CI: 87.4–92.9) among children above 5 years as compared to other age groups (p < 0.01). Comparing the seroprevalence levels by province—a similar pattern is observed throughout where type 1 and 3 seroprevalence was >90% and type 2 was significantly lower at 54.6%–68.8% (Fig. 3). There was no significant difference in seroprevalence between provinces that received 6 campaign rounds (Simbu, National Capital District and Central Province) and those that received 7 rounds (Madang, Morobe, Eastern Highlands).

**Fig. 4:**
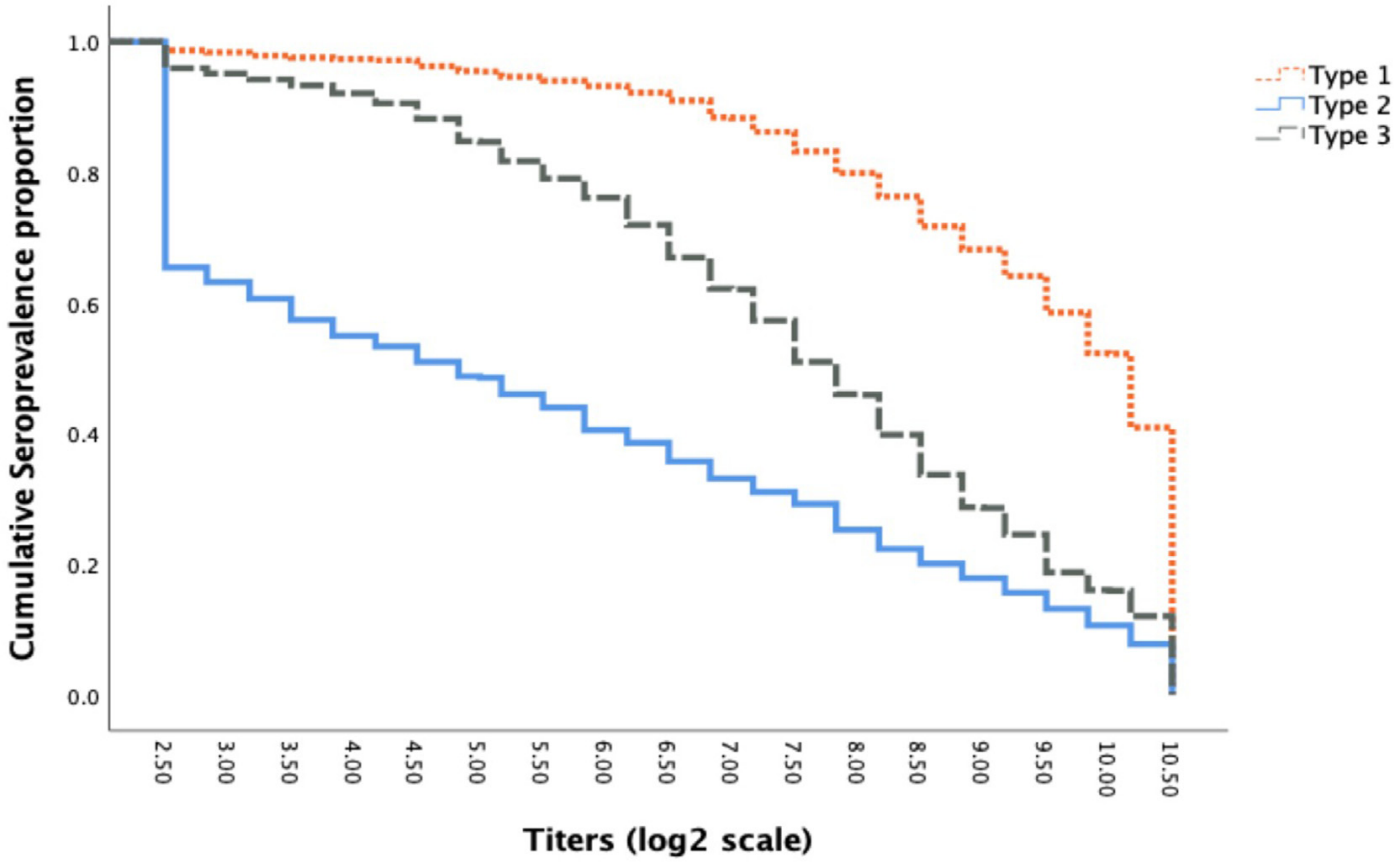
**Reverse cumulative distribution for all three poliovirus serotypes from 6 months to 10 years of age.** The results show significantly higher type 1 titres as compared to titres of types 2 and 3 (p < 0.01 each, respectively) and distribution of type 3 titre is significantly higher than type 2 titre distribution (p < 0.01).

The median titres in log_2_ scale with 95% bootstrap confidence interval is provided in Table 2 and the reverse cumulative titre distribution is shown in Fig. 4. Serotype 1 median titre was significantly greater than type 2 median titre in all age categories (p < 0.01). The reverse cumulative titre distribution also depicted very significantly high type 1 titres as compared to titres of types 2 and 3 (p < 0.01 each, respectively) and distribution of type 3 titre is significantly higher than type 2 titre distribution (p < 0.01). Of note, the titres for types 1 and 3 were high, but titres for type 2 were very low with median titres in the seronegative range.

**Table 2:** Median titres in log 2 scale for all polio serotypes with Bootstrap 95% CI by age categories.

Age categories	PV1	PV2	PV3
6 months–<1 year	≥10.5 (9.9–≥10.5)	<3 (<3–5.4)	9.5 (6.6–10.4)
1 year–<5 years	≥10.5 (10.2–≥10.5)	<3 (<3–4.2)	8.8 (7.5–10.2)
5 years–10 years	≥10.5 (9.5–≥10.5)	<3 (<3–4.2)	8.5 (7.2–9.8)

PV, poliovirus.

### Risk factor analysis

Children below the age of 1 year had significantly lower levels of seropositivity as compared to children above 5 years for all three serotypes (p < 0.01) (Table 3). Girls had higher rate of type 2 seropositivity as compared to boys (68.0% versus 58.5%, p < 0.01). Simbu Province had significantly lower levels of type 2 positivity compared to the other provinces (p < 0.01), however, the number of children who participated in the study from Simbu Province were very few. Children who had received OPV in SIA had a significant association with type 1 and 3 seropositivity (OR 13.02, 95% CI: 4.30–39.42, p < 0.01 and OR 3.08, 95% CI: 1.32–7.20, p = 0.01, respectively). Consuming protected water was significantly associated with type 2 seropositivity (p = 0.02) as compared to other water sources. Six bOPV SIAs were conducted in Central province, NCD, and Simbu. Seven bOPV SIAs were conducted in Eastern Highlands, Madang, and Morobe. There was no statistically significant difference in seroprevalence between provinces with different number of SIAs conducted (p = 0.07 for type 1, p = 0.06 for type 3).

**Table 3:** Association of selected characteristics with seroprevalence.

	N	SP1			SP2			SP3		
		%	n	p value	%	n	p value	%	n	p value
**Age groups**										
6 month–<1 year	53	86.8	46	<0.01	45.3	24	<0.01	81.1	43	<0.01
1 year–<5 years	488	99.2	484	0.64	40.4	197	<0.01	97.1	474	0.06
5 years–10 years	442	98.9	437		90.5	400		94.6	418	
**Sex**										
Female	487	97.9	477	0.32	68.0	331	<0.01	94.0	458	0.14
Male	496	98.8	490		58.5	290		96.2	477	
**Province**										
Central	151	100.0	151	0.39	67.5	102	<0.01	96.7	146	0.28
Eastern Highlands	226	98.2	222		54.9	124		95.1	215	
Madang	239	98.3	235		68.6	164		94.1	225	
Morobe	208	97.1	202		64.9	135		93.8	195	
National capital District	119	98.3	117		64.7	77		95.8	114	
Simbu	40	100.0	40		47.5	19		100.0	40	
**Site**^a^										
Low campaign areas	311	99.0	308	0.07	63.7	198	0.42	96.5	300	0.06
High campaign areas	692	97.4	674		62.7	434		93.4	646	
**Source of drinking water**										
Protected water	140	97.1	136	0.63	74.3	104	0.02	92.9	130	0.27
Pipe water	285	98.6	281		63.9	182		95.1	271	
Surface water	358	98.3	352		59.2	212		96.6	346	
Unprotected water	198	99.0	196		62.1	123		93.9	186	
**Defecation**										
Outside	967	98.3	951	<0.99	63.0	609	0.28	95.0	919	<0.99
Toilet	15	100.0	15		80.0	12		100.0	15	
**OPV received (in SIA)**^b^										
No	68	89.7	61	<0.01				88.2	60	0.01
Yes	912	99.0	903					95.7	933	
**Doses of OPV (among those received OPV in SIA)**										
1	64	98.4	63	0.41				95.3	61	0.08
2	151	98.0	148					92.1	139	
≥3	695	99.3	690					96.7	672	
**OPV in RI**										
1	147	93.9	138	0.11				87.1	128	0.13
2	92	98.9	91					95.7	88	
3	668	99.3	663					96.9	647	

OPV, oral poliovirus vaccine; RI, routine immunization; SIA, supplementary immunization activity; SP, seroprevalence.

Significance at p < 0.05.

a Low (6 campaigns): Central, NCD, Simbu. High (7 campaigns): Eastern Highlands, Madang, Morobe.

b Trend test.

## Discussion

PNG successfully interrupted transmission of cVDPV1 after several high coverage bOPV campaigns. Two years after the vaccination campaigns, the seroprevalence of poliovirus neutralizing antibodies to type 1 remained high (98%). Seroprevalence for type 1 was significantly lower in the youngest age group, 6–12 months which was born after the campaigns, compared to the older age groups, 1–4 and 5–10 years (88.5% versus 99% and 98.9%, respectively; p < 0.01).

All provinces in PNG received between 6 and 9 rounds of bOPV campaigns. There was no difference in seroprevalence between children from this study living in provinces where 6 or 7 rounds of bOPV campaigns were carried out, indicating that the value of additional vaccination campaigns is limited. The WHO Standard Operating Procedures for Outbreak Response outline that the vaccination response should include a rapid response round, followed by two large-scale rounds with a targeted mop-up round; further campaigns should be conducted if there is evidence of continued transmission.^14^ Documented cVDPV1 circulation in PNG stopped after two subnational and two national bOPV campaigns. The second national bOPV campaign in November 2018 targeted a wider age group from birth to 15 years. Expanding the age group and geography of vaccination response directly contributed to the interruption of virus transmission.^15^^,^^16^

For children born after April 2016, the only source of type 2 immunity in PNG has been through IPV in RI (WHO internal Polio Information Systems). The results from our study report uniformly low type 2 seroprevalence across all provinces (47.5%–68.6%). The eldest age group from the study (5–10 years) was born before the RI switch from trivalent to bivalent OPV; as such, type 2 seroprevalence in this age group was significantly higher at 90.5% (p < 0.01). Median titres were also higher in the older age group (7.83 versus <3 for both younger age groups, p < 0.0001). This was similarly documented in previous seroprevalence surveys in Beijing which sampled children before and after the tOPV to bOPV switch, and in Vietnam where IPV is shown to bridge the type 2 immunity gap.^17^^,^^18^ Girls had a higher rate of type 2 seropositivity as compared to boys (68.0% versus 58.5%, p < 0.01); this association hasn’t been seen in previous studies and we believe it may be incidental, and a study in East Sepik, PNG reveals no significant association between gender and vaccination status.^19^, ^20^, ^21^ Consuming protected water was significantly associated with type 2 seropositivity. This could suggest an area with increased sanitation and access to healthcare and a thus higher vaccination coverage in RI, however we do not have data to support this. Other studies highlight that good sanitation is an important factor in controlling the transmission by associating seropositivity with the type of toilet used, however we have not observed documented association with source of drinking water.^19^^,^^22^

There were several limitations to the study. SIA vaccination history suffers potential recall bias since SIAs are recorded based on parental reporting. RI is recorded in Children Health Books which were available in 69% (683/984) of participants. However, the recording tended to be incomplete. In particular, IPV vaccination history recording was significantly lacking. In addition, the study initiation coincided with the start of the COVID-19 pandemic which increased the complexity of study implementation in many ways. This resulted in samples being collected over a long (>1 year) period and the notably low enrolment rates from Simbu province were a result of fear and mistrust of visiting health teams coinciding with the introduction of COVID-19 vaccines.

This study further demonstrates the success of the country efforts to stop the cVDPV1 outbreak. Circulation stopped after high-quality nationwide bOPV campaigns, as supported by the high type 1 and 3 seroprevalence reported herein. However, the results are also useful to highlight the need to strengthen RI in PNG to prevent future outbreaks and emergences. The effect of SIAs will wane with time as new birth cohorts remain under-vaccinated. Moreover, the existing gap in type 2 seroprevalence poses risk of new cVDPV2 outbreaks if importation of the virus occurs. The emergence of cVDPV strains underscores the importance of maintaining high levels of RI coverage and effective surveillance systems for early detection.

## Contributors

WP, RF, VG, JG, TA, VJ, DB, DM, OM–protocol preparation, data collection, data verification, data analysis, manuscript preparation. KJ, BM–laboratory analysis, manuscript preparation. RLC, VJ, OM–data verification, data analysis, manuscript preparation.

## Data sharing statement

Unidentified data can be made available upon request. The full protocol can be requested for access after necessary country approvals for release are obtained from the corresponding author of this study, Rocio Lopez Cavestany (email: lopezro@who.int).

## Ethical approval

Obtained from the PNGIMR IRB, PNG MRAC and WHO Ethical Review Committee.

## Editor note

The Lancet Group takes a neutral position with respect to territorial claims in published maps and institutional affiliations.

## Disclaimer

The findings and conclusions in this report are those of the author(s) and do not necessarily represent the views of the Centers for Disease Control and Prevention and other contributing agencies.

## Declaration of interests

All authors—no conflict of interest declared.
