# Supplementary material for: Poliovirus serological assay after the cVDPV1 outbreak in Papua New Guinea: a cross-sectional study from 2020 to 2021
Source: Lancet Reg Health West Pac. 2023 Dec 20;44:100986. doi: 10.1016/j.lanwpc.2023.100986 (PMC10777103; doi:10.1016/j.lanwpc.2023.100986)
Supplement: Supplementary Materials [file mmc1.pdf]

Supplementary Materials: Table of Contents

Suppl 1: Sensitivity Analysis……………………………………………………………2

Suppl 1: Sensitivity Analysis

We have performed a sensitivity analysis to understand any potential bias in the sample population. The primary objective of the study was to assess the seroprevalence to types 1, 2 and 3 poliovirus to illustrate the efficacy of the outbreak response in Papua New Guinea. As part of the analysis, we associated vaccine history characteristics (OPV from RI and SIA) with seroprevalence level of type 1 and 3 polioviruses.

Using logistic regression, we found that seropositivity was 13x more likely amongst those who had received OPV in SIAs than those who had not. Moreover, children in RI who received ≥2 doses of OPV were significantly more likely to be seropositive than those who only received 1 dose ($p<0.05$) (**Table S1.1**).

The sensitivity analysis, which accounted for additional confounders (i.e., age, sex, and other demographic variables) yielded the same results (**Table S1.2**).

Sensitivity analysis was done only for types 1 and 3 poliovirus because bOPV was the vaccine administered in the campaigns (containing type 1 and 3). Type 2 seroprevalence would depend on IPV receiver through RI, however the recording in children health books was very limited as discussed in the manuscript, making this multivariable sensitivity analysis not reliable.

We have also assessed the average seroprevalence levels comparing the entire sample (n=1004), the analysed sample (n=984), and the excluded subjects (n=20) (**Table S1.3**). Children included for analysis were based on the protocol design of age between 6 months and 10 years. The estimates did not change even after the exclusion of the children outside of the age range.

Table S1.1: Association of vaccine history variables with types 1 and 3 seroprevalence, multivariate regression. OPV, oral poliovirus vaccine; OR, odds ratio; SP1/3, seroprevalence type 1/3

	**SP1**			**SP3**		
	**Prevalence OR**	**95% CI**	**P value**	**Prevalence OR**	**95% CI**	**P value**
**Received polio supplementary vaccine (OPV)**	13·02	4·30-39·42	<0·01	3·08	1·32-7·20	0·01
**OPV routine immunization doses received**						
**1**						
**2**	9·68	1·13-83·02	0·04	3·71	1·20-11·51	0·02
**≥3**	7·91	2·42-24·79	<0·01	4·62	2·38-8·94	<0.01

Table S1.2: Association of vaccine history variables with types 1and 3 seroprevalence adjusted for confounders, multivariate regression. OPV, oral poliovirus vaccine; OR, odds ratio; SP1/3, seroprevalence type 1/3

	**SP1**			**SP3**		
	**Prevalence OR**	**95% CI**	**P value**	**Prevalence OR**	**95% CI**	**P value**
**Received polio supplementary vaccine (OPV)**	13.02	4.30-39.42	<0.01	1.24	0.36-4.32	0.73
**OPV routine immunization doses received**						
**1**						
**2**	9.68	1.13-83.02	0.04	4.14	1.27-13.50	0.02
**≥3**	7.91	2.42-24.79	<0.01	4.14	2.10-8.16	<0.01
**Age categories**[a]						
**6 months to <1 year**	0.24	0.03-1.81	0.17	0.30	0.09-0.98	<0.05

**1 year to <5 years**	1.89	0.42-8.65	0.41	2.08	0.97-4.44	0.06
**5 years to 10 years**						
**Gender**						
**Male**	2.05	0.62-6.72	0.24	1.77	0.91-3.44	0.09
**Site[a]**						
**Low campaign areas**						
**High campaign areas**	0.43	0.09-2.05	0.29	0.64	0.30-1.35	0.24

Table S1.3: Average seroprevalence for all three poliovirus types. N1=There were 1006 children included in the study, of which 1004 serology results. N2=Included in the final analysis were 984 children (exclusions based on outside of age range). N3=There were 22 children that were excluded from analysis based on age, of which 21 had serology results. PV1/2/3, poliovirus type 1/2/2; 95% CI, 95% confidence interval.

	Sample	Seroprevalence	95% CI
**PV1**	N1=1004	97.9	96.3-95.7
	N2=984	98.3	97.4-98.9
	N3=21	76.2	52.8-91.8
**PV2**	N1=1004	62.9	59.9-65.9
	N2=984	63.1	60.1-66.1
	N3=22	52.5	29.8-74.3
**PV3**	N1=1004	94.3	92.7-95.7
	N2=984	95.0	93.6-96.3
	N3=21	57.1	34.0-78.2
